# Surface Proteins of *Staphylococcus epidermidis*

**DOI:** 10.3389/fmicb.2020.01829

**Published:** 2020-07-29

**Authors:** Timothy J. Foster

**Affiliations:** Department of Microbiology, Trinity College Dublin, Dublin, Ireland

**Keywords:** biofilm, amyloid structures, bacterial adhesion, extracellular matrix, homophilic protein-protein interaction, MSCRAMM, phylogenetics

## Abstract

*Staphylococcus epidermidis* is a ubiquitous commensal of human skin. The widespread use of indwelling medical devices in modern medicine provides an opportunity for it to cause infections. Disease causing isolates can come from many different genetic backgrounds. Multiply antibiotic resistant strains have spread globally. *S. epidermidis* has a smaller repertoire of cell wall anchored (CWA) surface proteins than *Staphylococcus aureus*. Nevertheless, these CWA proteins promote adhesion to components of the extracellular matrix including collagen, fibrinogen, and fibronectin and contribute to the formation of biofilm. The A domain of the accumulation associated protein Aap can promote adhesion to unconditioned biomaterial but must be removed proteolytically to allow accumulation to proceed by homophilic Zn^2+^-dependent interactions. Mature biofilm contains amyloid structures formed by Aap and the small basic protein (Sbp). The latter contributes to the integrity of both protein and polysaccharide biofilm matrices. Several other CWA proteins can also promote *S. epidermidis* biofilm formation.

## Introduction

*Staphylococcus epidermidis* is a ubiquitous and primarily harmless commensal of human skin compared to the more pathogenic coagulase-positive *Staphylococcus aureus* (Becker et al., 2014). Modern medicine allows the successful treatment of patients with serious and potentially life-threatening illnesses. Indwelling medical devices such as intravenous catheters, prosthetic joints, and heart valves provide an opportunity for *S. epidermidis* to gain access to the body and to cause infections.

The human skin is acidic, dessicated, nutrient poor and has high osmolarity. Shotgun metagenomic whole genome sequencing has allowed the relative abundance of bacteria at different skin sites to be established (Byrd et al., 2018). *S. epidermidis* is a prominent member of the microbiome of both dry and moist skin, as well as areas with sebaceous glands. It is also an important component of the nasal microbiome (Byrd et al., 2018; Liu et al., 2020). There is growing awareness that *S. epidermidis* is not just a benign resident but rather has a proactive role in modulating the host immune system to promote survival of commensals and influence the development of the skin and nasal microbiome (PrabhuDas et al., 2011; Naik et al., 2012, 2015).

Phylogenetic analysis of a large number of commensal strains of *S. epidermidis* isolated from the skin of healthy humans and nosocomial disease-causing isolates indicates that the species comprises two discrete clusters called A/C and B (Meric et al., 2018). Defining the core genome and the large and growing number of accessory genes indicates that *S. epidermidis* is open to horizontal gene transfer of mobile genetic elements and to chromosomal recombination. Organisms in the A/C cluster are more likely to cause nosocomial infections while the B cluster strains have a higher proportion of commensals.

The two groups are adapted to colonize distinct habitats on the skin and are genetically isolated. A/C strains are adapted to colonize the surface of skin and are fitter under acidic and osmotic stress, they grow well at pH 4.5 typical of macrophage and neutrophil phagosome acidification, and they form biofilm at pH7 (the pH of blood; Espadinha et al., 2019). B strains survive in the microaerophilic environment of deeper skin sites, they are more resistant to bactericidal fatty acids and they metabolize lipids found in sebaceous glands and hair follicles (Espadinha et al., 2019).

*S. epidermidis* isolated from 15 different body sites ranging from the face to the toe web was subjected to whole genome sequencing. Material from swabs was also analyzed by metagenomic sequencing (Zhou et al., 2020). This revealed that an individual is colonized by multiple lineages whose genomes have diversified by mutation and horizontal gene transfer. A broad representation of phylogenies from both A/C and B groups was found at most sites. Frequent transmission of bacteria between body sites was evident (e.g., face to hand). In contrast, *S. epidermidis* from the toe web showed little diversification suggesting that this is an isolated niche.

Organisms from both clades can cause infection. Genome wide association studies of commensal and disease isolates identified infection-associated genetic sequences in loci associated with biofilm formation, toxicity, inflammation, and resistance to antibiotics (Meric et al., 2018). Whole genes and short sequences carrying allelic variations within genes have been transmitted by chromosomal recombination, while entire SCC*mec* elements encoding β-lactam resistance and potential virulence determinants (Qin et al., 2017; Arora et al., 2020) have been transmitted horizontally. It was concluded that many strains from the two clades can cause disease.

In contrast, three distinct hospital-adapted clones from clonal complex 2 have spread globally (Lee et al., 2018). These strains are resistant to multiple antibiotics including β-lactams and rifampicin. The *rpoB* mutations that confer resistance to rifampicin were analyzed genetically and were found to contribute to insensitivity to the glycopeptide vancomycin. A small proportion of cells within the population expresses a low but clinically significant level resistance to vancomycin and will outgrow the majority in the presence of the drug to compromise the treatment of patients. A toxin expressed by the SCC*mec* element contributes to the pathogenesis of sepsis (Qin et al., 2017).

Biofilm formation on the surface of indwelling medical devices is a major virulence attribute. Two distinct mechanisms of biofilm accumulation have been identified. One requires formation of a polysaccharide glycocalyx and the other involves cell wall anchored (CWA) surface proteins.

*S. epidermidis* possesses a smaller repertoire of virulence factors compared to *S. aureus*. It lacks the immune evasion proteins and cytolytic toxins that are characteristics of *S. aureus*. It can express several CWA surface proteins including proteins that promote biofilm formation and bind components of the extracellular matrix such as fibronectin, collagen, and fibrinogen (Table 1). Their properties will be discussed in the context of their contribution to biofilm formation and the pathogenesis of implanted device-related infections.

**Table 1:** Functions of surface-associated proteins.

Protein	Ligands	Role in biofilm formation
SdrF	A and B domains bind collagen	Adhesion to collagen deposited on *ex vivo* biomaterial Adhesion to abiotic surfaces
SdrG/Fbe	A domain binds fibrinogen	Promotes adhesion to conditioned biomaterial
Aap	None reported. G5-E repeats engage in homophilic interactions	Adhesion to abiotic surfaces *via* A domain. Cell-cell accumulation. Amyloid formation in mature biofilm.
Embp	FIVAR and FIVAR-GA domains bind fibronectin	Adhesion to conditioned biomaterial? FIVAR-GA region binds unknown ligand(s) on adjacent cells to promote accumulation
SdrC	Not known	Biofilm formation by unknown mechanism
SesJ	Plasminogen	Not known
SesI	Not known	Might promote adhesion to abiotic surfaces. Otherwise unknown
Geh	Collagen	Pure Geh binds collagen *in vitro*. Bacterial adhesion to immobilized collagen not tested
AtlE	Autolysin Recombinant protein binds vitronectin, fibronectin and fibrinogen *in vitro*	Release of e-DNA promotes adhesion to abiotic surfaces Adhesion to conditioned biomaterial?
Sbp	Secreted protein. Associated with cell wall. Possibly binds Aap	Amyloid formation contributes to biofilm integrity

SdrF, SdrG/Fbe, Aap, Embp, SdrC, SesJ, and SesI are anchored covalently to peptidoglycan by sortase.

## Cell Wall Anchored Surface Proteins

### Steps in Biofilm Formation

The ability to form biofilm on indwelling medical devices is crucially important and is a major virulence determinant of *S. epidermidis*. The focus of this review is the function of CWA proteins in this process (Table 1). Many clinical isolates can express both polysaccharide and protein based mechanisms of biofilm formation depending on *in vitro* growth conditions.

The hallmark of infections associated with indwelling devices is the ability of bacteria to adhere to the implant and to grow as a biofilm (Otto, 2018). Biofilm formation is initiated by attachment of bacteria to the surface of the biomaterial, either to an abiotic surface prior to or at the time of implantation, or to a surface that has been conditioned by deposition of host proteins. Attachment is followed by the accumulation phase where bacteria multiply to form multicellular communities. This requires the cells to stick to each other by mechanisms that promote intercellular adhesion. The biofilm undergoes maturation with the creation of channels by release of some cells in the matrix. In devices that are in contact with the bloodstream detached cells can disseminate.

Investigation of the mechanisms involved in biofilm formation by *S. epidermidis* has been carried out with a small number of strains that could be manipulated genetically. This has mainly involved studies of biofilm formation *in vitro* under static growth conditions and dynamically in flow chambers allowing biofilms to be visualized and quantified by confocal microscopy. *In vitro* studies were complemented with *in vivo* models involving catheter segments implanted subcutaneously or intravenously in rodents.

### Adhesion to Abiotic Surfaces

Investigation into the mechanistic basis of biofilm formation by *S. epidermidis* began with the analysis of biofilm defective transposon insertion mutants of strain O-47 (Heilmann et al., 1996). Some mutations knocked out expression of the autolysin AtlE (Heilmann et al., 1997). The mutant was defective in attachment to a polystyrene surface. However, it is unclear if the AtlE protein itself is an adhesin because a null mutation affecting a major enzyme involved in cell wall metabolism results in pleiotropic changes to the cell surface. Point mutations or short in-frame deletions that are defective in adhesion while remaining enzymatically active are required.

The presence of DNase reduced adhesion of both clinical isolates and laboratory strains 1457 and RP62a to plastic and glass surfaces under static and hydrodynamic conditions (Qin et al., 2007). An AtlE mutant of strain 1457 produced much lower levels of extracellular (eDNA). It was concluded that release of eDNA from a small number of cells in the population by the autolytic activity of AtlE promotes bacterial attachment to abiotic surfaces and is important in the primary attachment phase of biofilm formation. Thus, the role of AtlE may be to produce eDNA rather than itself acting as an adhesin.

Adhesion to the abiotic surface plastic biomaterial can also be promoted by the cell wall-associated proteins SdrF (Arrecubieta et al., 2009) and the accumulation associated protein Aap (Conlon et al., 2014). Genomic analysis of *S. epidermidis* revealed the presence of a gene encoding a CWA protein with homology the biofilm associated protein Bap of *S. aureus* (Bhp, Bap homologous protein; Bowden et al., 2005). However, while Bap promotes both attachment to abiotic surfaces and biofilm accumulation (Cucarella et al., 2001), nothing is known about Bhp.

**Figure 1:**
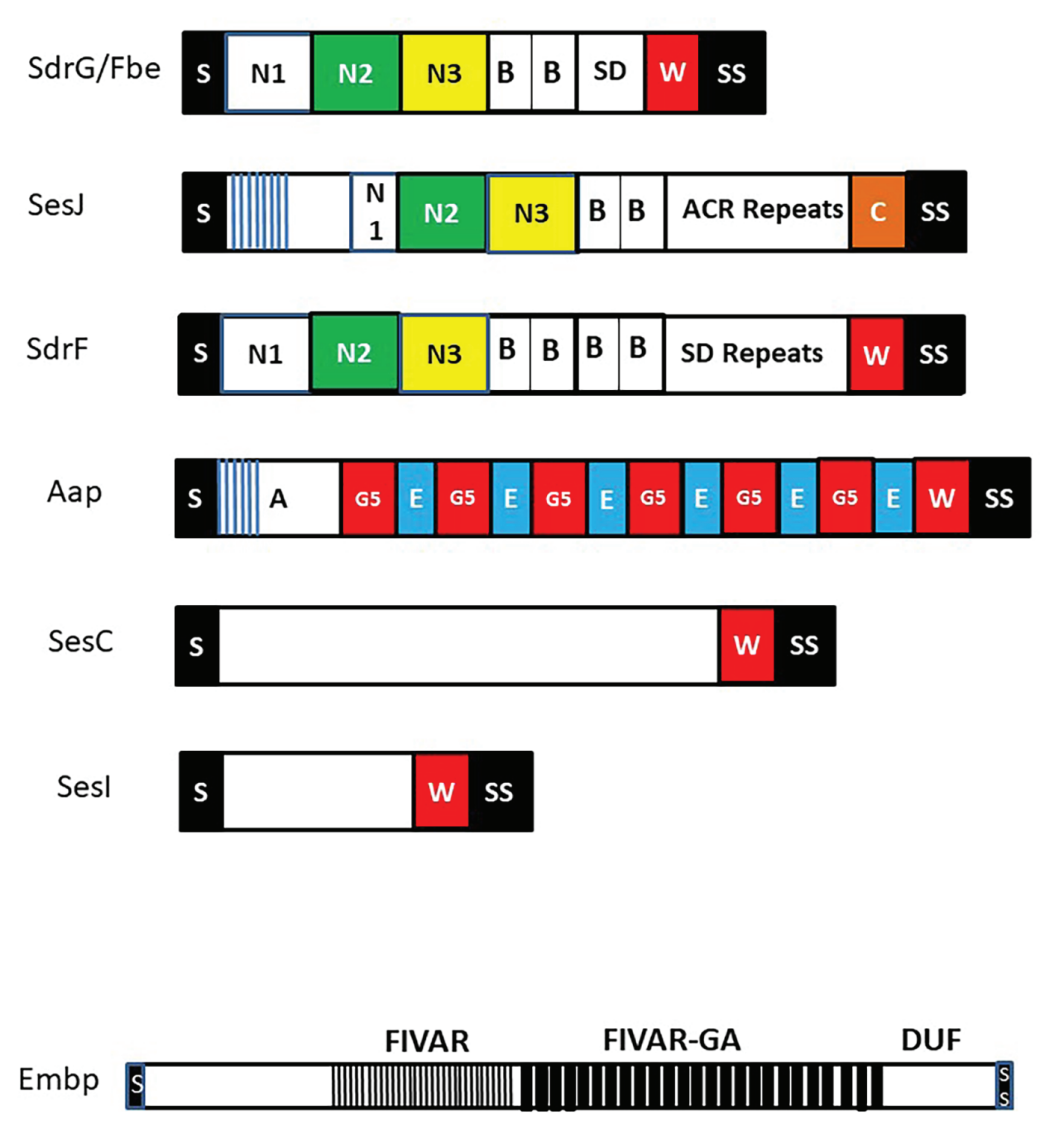
Cell wall anchored (CWA) surface proteins of *Staphylococcus epidermidis*. Schematic diagram of CWA surface proteins that feature in the text. Each protein has a secretory signal (S) at the N-terminus that is removed during secretion and at the C-terminus a sorting signal (SS) that promotes covalent anchorage to cell wall peptidoglycan. Many CWA proteins have a proline-rich cell wall spanning domain W while SesJ has a collagen-like sequence C. Extracellular matrix binding protein (Embp) is shown separately to emphasize the order of magnitude greater size compared to the other proteins. It contains several domains of unknown function (DUF) C-terminal to the found in various architectures (FIVAR) and FIVAR–G-related albumin binding (FIVAR-GA) repeats. Proteins in the microbial surface components recognizing adhesive matrix molecules (MSCRAMM) family are shown at the top with the canonical N2 and N3 subdomains that have potential to engage ligands by the dock lock latch mechanism. Each contains separately folded B repeat domains and unfolded serine asparate (SD) repeats or in the case of SesJ a distinct aspartate containing region (ACR). The SD repeat region of SdrG from strain RP62a is very short. Its length will likely vary in other strains. The N-termini of the A domains of SesJ and Aap contain distinct short N-terminal repeats (blue lines).

SdrF is a member of the microbial surface components recognizing adhesive matrix molecules (MSCRAMM) family of CWA proteins with an N-terminal A domain linked to B repeats (Figure 1). The archetypal MSCRAMM SdrG/Fbe is described below. SdrF promotes adhesion to the highly textured surface of the hydrophobic polymer Dacron used to coat the drive lines of ventricular assist devices (VAD) and could provide *S. epidermidis* with a portal for entering and colonizing the indwelling driveline (Arrecubieta et al., 2009). This was investigated using the non-adhesive surrogate host *Lactococcus lactis* expressing full length SdrF and truncates expressing the A domain or the B domains alone. Both domains promoted adhesion which could be blocked using domain-specific antibodies.

### Adhesion to Conditioned Biomaterial

Newly implanted indwelling devices are rapidly coated with host plasma proteins such as fibronectin and fibrinogen (Vaudaux et al., 1995). The conditioning layer of long term implants such as a VAD drive lines also contains collagen (Arrecubieta et al., 2009).

#### SdrG/Fbe Binds Fibrinogen

The SdrG/Fbe protein is CWA protein of the MSCRAMM family (Figure 1; Foster et al., 2014; Foster, 2019). The A domain binds to the C-terminus of the β-chain of fibrinogen in the central E region. X ray crystallography of the A domain in the unbound apo form and in complex with the β-chain fibrinopeptide allowed formulation of the “dock lock latch” mechanism of ligand binding (Ponnuraj et al., 2003). A key feature of the binding mechanism is that it is facilitated by and strengthened by shear stress (Milles et al., 2018). Forces equivalent to those required to break a covalent bond are required to separate SdrG from its bound ligand (Herman et al., 2014).

Binding to immobilized fibrinogen/fibrin in the conditioning layer of an indwelling device is important in initiating device-related infections. *S. epidermidis* HB adhered to immobilized fibrinogen *in vitro* in a SrdG/Fbe-dependent manner (Hartford et al., 2001; Pei and Flock, 2001). Expression of the MSCRAMM was required for the bacterium to colonize the surface of a catheter implanted intravenously in rats (Guo et al., 2007).

#### SdrF Binding to Collagen

In addition to promoting adhesion to abiotic surfaces, SdrF also promotes bacterial adhesion to immobilized type I collagen (Arrecubieta et al., 2007). This could be important in VAD driveline infections where the conditioning layer contains collagen (Arrecubieta et al., 2009). Adhesion of *S. epidermidis* to *ex vivo* drivelines was in part promoted by SdrF. Atomic force microscopy showed that both the A domain and B repeats could bind collagen (Herman-Bausier and Dufrene, 2016). These interactions were weak in contrast to the strong binding that is characteristic of the collagen binding protein Cna of *S. aureus* binding collagen by the hug mechanism (Zong et al., 2005; Herman-Bausier et al., 2016; Foster, 2019).

#### Does SesC Promote Binding to Fibrinogen?

Expression of the CWA surface protein SesC ectopically from a multicopy plasmid in a low fibrinogen binding strain of *S. aureus* and in *S. epidermidis* RP62a resulted in a slight but significant increase in bacterial adherence to immobilized fibrinogen (Shahrooei et al., 2009). However, recombinant SesC protein did not bind Fg *in vitro*, so the affinity and specificity of the interaction could not be measured. Therefore, the suggestion that SesC promotes binding to Fg must be treated with caution. The role of SesC in biofilm formation is discussed below.

#### Embp Binds to Fibronectin

*S. epidermidis* expresses a very large (10,204 residue) CWA surface protein called the extracellular matrix binding protein (Empb; Williams et al., 2002; Christner et al., 2010). A major part of the protein comprises two long repeated domains called found in various architectures (FIVAR, *n* = 21) and FIVAR–G-related albumin binding (FIVAR-GA, *n* = 38; Figure 1). Overexpression of Embp by *S. epidermidis* promoted bacterial adhesion to immobilized fibronectin. Recombinantly expressed FIVAR and FIVAR-GA modules both bound Fn in solid phase ELISA-type binding assays with similar profiles. Surface plasmon resonance indicated a binding affinity in the nanomolar range. Embp expression was induced by growth in serum, so it is possible that the protein contributes to adhesion to conditioned biomaterial *in vivo*.

#### Autolysins and Other Surface Associated Proteins

It has been suggested that autolysins, AtlE and Aae, promote *S. epidermidis* adhesion to immobilized plasma proteins vitronectin, fibronectin, and fibrinogen and that they could be involved in initiating biofilm formation on conditioned biomaterial surfaces (Heilmann et al., 1997, 2003). However, these binding studies were only performed with purified recombinant proteins and must be interpreted with caution. The involvement of autolysins in promoting bacterial adhesion to immobilized ligands was not investigated. An AtlE mutant had reduced virulence in a rat intravenous catheter infection model but this could be attributed to pleiotropic effects and lack of fitness due to loss of the cell wall metabolizing autolysin (Rupp et al., 2001).

#### Gycerol Ester Hydrolase

The glycerol ester hydrolase (Geh) can bind to collagen *in vitro* (Bowden et al., 2002). However, any role in promoting bacterial adhesion during biomaterial associated infection remains speculative.

### Biofilm Accumulation

Following attachment to surfaces bacteria multiply and form multicellular aggregates requiring cells to adhere to each other (Becker et al., 2014; Otto, 2018). Intercellular adhesion can be promoted by the polysaccharide intercellular adhesin (PIA) or by surface proteins. The extracellular matrix of a mature biofilm contains DNA and proteins released from lysed cells. Biofilm formed *in vivo* will also harbor host proteins.

#### Polysaccharide Intercellular Adhesin

In many strains, the molecule responsible for accumulation phase of biofilm is PIA, a homopolymer comprising at least 130 units of β-1-6-linked N-acetylglucosamine (PNAG; Mack et al., 1996; reviewed by Otto, 2018). The polysaccharide chains are synthesized intracellularly by the integral membrane proteins IcaA and IcaD (Mack et al., 1996; Vuong et al., 2004a). The polysaccharide is transported across the membrane by IcaC. The extracellular polysaccharide is partially (15–20%) deacylated by the IcaD protein. This is essential for biofilm formation because the exposed positively charged NH^3+^ groups allow the molecule to attach by electrostatic interactions to the negatively charged bacterial cell surface (Vuong et al., 2004a). However, the molecules involved in binding PNAG to the cell surface have not been identified. This process is likely to be multifactorial; a mutant defective in wall teichoic acid still carried PNAG on its surface and formed biofilm *in vitro* (Vergara-Irigaray et al., 2008).

The presence of PIA on the cell surface is an important immune evasion mechanism (Le et al., 2018) both because aggregates inhibit engulfment by phagocytes and also because it acts as a capsule and inhibits opsonophagocytosis (Vuong et al., 2004b). Biofilm formation *in vivo* by an Ica^+^ strain induced lower levels of proinflammatory cytokines than an Ica^−^ mutant (Fredheim et al., 2011). Ica strongly activated complement but paradoxically reduced the activation of phagocytes contributing to reduced eradication of biofilm. Inflammation adjacent to an infected catheter insertion site was more severe with a wild type strain (Kristian et al., 2008). Ica also protected against antimicrobial peptides (Vuong et al., 2004b).

The *icaADBC* genes form an operon which is negatively regulated by the IcaR repressor protein. Global transcriptional regulators SigB and SarA, as well as the quorum sensing system LuxS also contribute to the regulation of *ica* expression. The environmental signals that regulate *ica in vivo* are complex and not well understood (O’Gara, 2007).

#### Accumulation Associated Protein

Device related infections can frequently be caused by strains of *S. epidermidis* that lack the ability to form PNAG/PIA (Kogan et al., 2006; Rohde et al., 2007). Indeed, the *ica* genes are only present in 37% of lineage A–C strains and 4% of group B strains (Espadinha et al., 2019) and one third of disease isolates lack *ica* (Rohde et al., 2007). In contrast, the gene that encodes the CWA accumulation associated protein Aap is widespread in clinical isolates (Kogan et al., 2006; Petrelli et al., 2006; Rohde et al., 2007; Hellmark et al., 2013).

Aap promoted biofilm formation by strain 1457 *in vitro* under static and dynamic conditions and it promoted colonization of a catheter implanted into the jugular vein of rats (Schaeffer et al., 2015). It contributed to biofilm formation *in vivo* either alone in strains that lack *ica* or in strains that also express PIA/PNAG.

**Figure 2:**
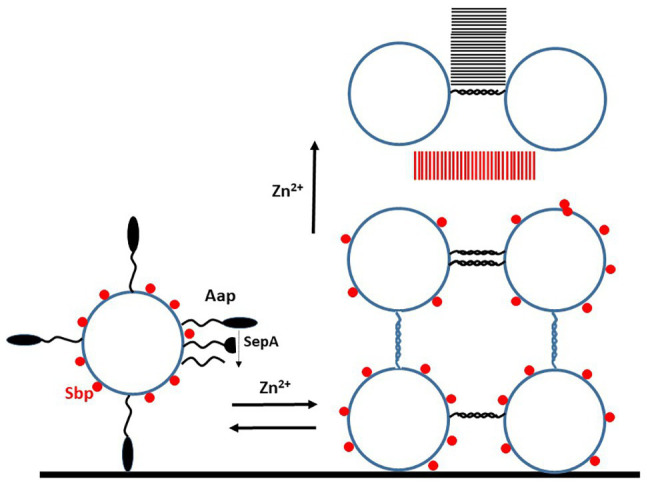
Mechanism of biofilm formation promoted by Aap. Schematic diagram of events that occur during biofilm formation by *S. epidermidis* strains expressing Aap. The intact A domain of the Aap protein can promote attachment to unconditioned (abiotic) biomaterial. In order to participate in the accumulation stages, the A domain must be removed either completely or partially by protease. The small basic protein (Sbp) is associated non-covalently with the cell wall. The first phase of biofilm formation is Zn^2+^-dependent and reversible by adding a chelator. This involves homophilic interactions between G5-E repeats on adjacent cells. As the biofilm matures Sbp and the Aap proteins form amyloid fibrils (parallel lines), in the case of the latter this is Zn^2+^-dependent but irreversible. Sbp is also implicated in biofilm formation involving PIA.

The N-terminal A domain of Aap promoted attachment to abiotic surfaces (Conlon et al., 2014). The A domain comprises two subdomains, the N-terminal repeats followed by a lectin-like domain (Figure 1). The A domain must be removed either completely or in part by proteolysis allowing the exposed C-terminal G5-E domains to participate in biofilm accumulation (Figure 2; Rohde et al., 2005; Paharik et al., 2017). The metalloprotease SepA has two cleavage sites in Aap, one is located between the N-terminal repeats and the lectin-like domain while the other occurs between the A domain and the G5E repeats. Aap isolated from the surface of planktonic cells and from a mature biofilm matrix comprises a mixture of fully and partially cleaved proteins (Rohde et al., 2005; Yarawsky et al., 2020).

The Aap protein of *S. epidermidis* is very similar to the SasG protein of *S. aureus* (Roche et al., 2003). Conclusions drawn from studies with Aap can most likely be applied to SasG and vice versa. Each C-terminal B repeat domain of Aap and SasG comprises two segments called G5 and E (Gruszka et al., 2012, 2015; Conrady et al., 2013). The regions have unusual properties allowing them to fold efficiently to form extended fibrils that are very stable. The E and G5 domains are potentially intrinsically disordered. The minimum unit capable of correct folding is G5-E-G5.

Biofilm accumulation promoted by Aap occurs in two stages following cleavage of the A domain (Figure 2). First Zn^2+^-dependent homophilic interactions occur between exposed G5E repeat domains on adjacent cells that result in twisted rope-like structures (Conrady et al., 2013). This interaction occurs *in vitro* with purified G5-E repeat proteins in solution. Atomic force microscopy studies performed with *S. aureus* expressing SasG showed that considerable force is required to separate cells held together by fully engaged proteins (Formosa-Dague et al., 2016). This interaction could be reversed by addition of the chelator DTPA reflecting the Zn^2+^ dependence of recombinant protein-protein interactions *in vitro* and Aap-promoted bacterial biofilm formation.

The second stage is assembly of G5-E repeats into higher order amyloid structures in a Zn^2+^− dependent fashion. This was investigated by sedimentation analysis (Yarawsky et al., 2020). Amyloids were visualized by transmission electron microscopy both with recombinant G5-E proteins in solution and in a bacterial biofilm matrix formed by strain RP62a *in vitro*. Mass spectroscopy analysis of tryptic digests of purified aggregates isolated from biofilm detected peptides from the lectin domain of region A indicating that complete removal of the A domain is not required for amyloid formation. The amyloids could not be reversed by the chelator DTPA, which is consistent with resistance of established biofilms to dissociation by the chelator.

#### Small Basic Protein

A small (18 kDa) basic protein called Sbp was recently shown to have an important role in biofilm (Decker et al., 2015). In solution, Sbp is monomeric and partially unfolded. Following agitation recombinant, Sbp forms multimeric protein complexes – amyloid fibrils – that can be stained with the fluorescent dyes thioflavin S and Congo red (Wang et al., 2018).

The role of Sbp in biofilm formation was studied in strain 1457 that forms robust biofilm incorporating both PIA/PNAG and Aap (Decker et al., 2015). Despite being a secreted soluble protein, Sbp was isolated predominantly from the cell wall fraction rather than in the culture supernatant of planktonic bacteria growing under biofilm forming conditions. Confocal microscopy of biofilm showed that Sbp forms large clumps (possibly amyloids) that are unevenly dispersed and concentrated at the surface of the cell aggregates. By studying isogenic mutants, it was shown that Sbp is not required for primary attachment of cells to unconditioned biomaterial but that it is required for mechanically robust tethering of mature biofilm. Biofilm formed statically by an Sbp mutant was more easily washed away. This is consistent with much lower levels of biofilm formed by the mutant under dynamic shear stress. However, *in vivo* a lack of Sbp did not affect the density of biofilm formed on subcutaneously implanted catheters but it should be noted that this model involves injecting bacteria into the lumen of the catheter where it is unlikely that bacteria would be subjected to shear stress. It would be interesting to test the mutant in the rat jugular vein catheter model where bacteria colonize the indwelling device following haematogenous spread.

Contradictory data have been published about the ability of Sbp to interact with the G5-E repeat region of Aap. In solution, no interaction was detected between recombinant Sbp and the minimum folded and functional Aap G5-E-G5 protein (Wang et al., 2018). In contrast, when Sbp was subjected to SDS-PAGE and electroblotting, it showed binding to the soluble full length G5-E repeat protein. In an ELISA based assay, soluble Sbp bound to immobilized G5-E repeats in a dose-dependent and specific manner (Decker et al., 2015). These observations led to the conclusion that Sbp contributes to the biofilm structure by interaction with the G5-E repeats of Aap. Sbp is clearly of importance in the formation of and integrity of biofilms most likely because of its innate ability to form amyloids. Whether or not it interacts with the G5E-repeats of Aap is a lesser consideration.

#### SesC and Biofilm Formation

The possible role of SesC in biofilm formation by *S. epidermidis* was investigated by testing the inhibitory effect of anti-SesC IgG *in vitro* and *in vivo* and by ectopic expression of the protein in *S. aureus* (Shahrooei et al., 2009, 2012; Khodaparast et al., 2016). Anti-SesC IgG reduced both the primary attachment and accumulation phases of biofilm formation *in vitro*. However, the suggestion that SesC has a direct role in these events must be treated with a degree of caution since the presence of IgG bound to a protein on the bacterial cell surface could have pleiotropic inhibitory effects. The clearest evidence for a direct role was the enhanced protein-dependent biofilm formed *in vitro* and *in vivo* by 8325-4 expressing SesC (Khodaparast et al., 2016). Further studies with *sesC* null mutants are required.

#### Embp and Biofilm Formation

Overexpression of the giant surface protein Embp *in vitro* by a clinical isolate of *S. epidermidis* that lacks both *ica* and *aap* genes led to clustering of planktonic cells and to biofilm formation (Christner et al., 2010). Embp did not promote primary attachment to abiotic surfaces but rather biofilm accumulation. The mechanistic basis of aggregation seems to involve heterophilic interactions between the repeated FIVAR-GA domains and ligands on the surface of adjacent cells. Recombinant FIVAR-GA but not FIVAR domains promoted aggregation of cells that lacked the ability to express Embp. Inhibition of Embp-promoted biofilm by antibody directed against both FIVAR-GA or FIVAR domains could be interpreted as the specific blocking of heterophilic interactions in the case of the former and indirect blocking with the latter.

### Biofilm Maturation

As the biofilm develops it undergoes maturation to form three-dimensional structures with mushroom-like towers and fluid filled gaps (Otto, 2018). Small amphipathic α-helical peptides with surfactant properties called phenol soluble modulins (PSMs) disrupt non-covalent bonds formed between cells during biofilm development to promote the formation of channels (Le et al., 2019). Bacterial cells that are released from the maturing biofilm *in vivo* enter the bloodstream causing bacteraemia. Mutants of laboratory strain 1457 lacking the ability to express PSMs formed unstructured biofilms under both static and flow conditions *in vitro* that lacked channels and had a greater mass (Wang et al., 2011; Le et al., 2019). In a mouse model of subcutaneous catheter-associated biofilm infection, the *psm* null mutant formed a more substantial biofilm but was disseminated less effectively. This is consistent with the notion that PSMs promote biofilm maturation and structuring.

There is some debate as to whether PSMs contribute to the biofilm matrix by forming amyloid structures (Schwartz et al., 2012). Some of the PSMs expressed by *S. aureus* have the ability to form amyloids *in vitro* (Schwartz et al., 2012) but none of the *S. epidermidis* PSMs displayed that property (Le et al., 2019). Enhanced resistance of wild type *S. aureus* biofilm to degradation by DNase compared to a PSM-defective mutant that was interpreted as being a reflection of PSM amyloids (Schwartz et al., 2012) but could be the result of direct binding of PSMs to DNA (Zheng et al., 2018).

The *psm* genes are directly regulated by the Agr quorum sensing system (Queck et al., 2008), and PSM expression is likely to be induced in the cells that are closely packed together in a biofilm matrix. Blocking Agr has been proposed as an anti-virulence strategy (Dickey et al., 2017). In the case of *S. epidermidis*, this may not influence the formation of a biofilm on an indwelling medical device but could instead reduce bacteraemia, dissemination, and sepsis.

### Other Cell Wall Anchored Proteins

Surface proteins that have been shown to have a role in biofilm formation have been discussed in the previous section. Two other CWA proteins expressed by *S. epidermidis* with possible roles in the pathogenesis of bloodstream infection have been investigated.

#### SesJ

The majority of genes encoding members of the MSCRAMM family of CWA proteins of *S. epidermidis* are present in the core genome (Conlan et al., 2012). A notable exception is *sesJ* which is carried by SCC*mec* or ACME mobile genetic elements (Arora et al., 2020). SesJ was present in about 18% of clinical isolates from bloodstream infections collected in two US hospitals.

SesJ is a chimaeric protein that is a member of a subfamily of MSCRAMMs (Figure 1; Arora et al., 2016). Members occur in other CoNS including SdrI in *S. saprophiticus* (Sakinc et al., 2006). The protein has an N-terminal repeat region comprising 15 residues repeated 13–15 times. This is reminiscent of the NTR region of Aap although they lack any sequence similarity. The A region N2 and N3 subdomains have all of the features of a typical MSCRAMM including a TYTFTDYVD motif and a putative latching peptide. However, the N1 subdomain is shorter than that of SdrG and SdrF. The A region occurs in two isoforms that are 95% identical (Arora et al., 2020). This is followed by two B repeats which are similar in sequence and predicted structure to the B repeats of SdrG and SdrF. Linking the A and B regions to the cell wall are aspartate containing repeats, in contrast to the serine aspartate dipeptide repeats of typical MSCRAMMs (Foster et al., 2014; Foster, 2019).

The *sesJ* gene is accompanied by two genes encoding glycosyltransferases (Arora et al., 2020). The gene encoding isoform 1 SesJ is located in an SCC*mec* typeIV element while the isoform 2 gene is located within an ACME element. These were found mainly in ST2, ST5, and ST210 strains.

Studies to identify the ligand(s) recognized by SesJ have been inconclusive (Arora et al., 2020). The recombinant A domain did not bind to fibrinogen, fibronectin, laminin, vitronectin, and several types of collagen. It did, however, bind plasminogen although the involvement of the DLL mechanism was not investigated. *S. aureus* expresses several CWA proteins that bind plasminogen (Pietrocola et al., 2016). The best characterized is FnBPB where the A domain binds *via* ionic bonds between surface located lysine residues and kringle domain 4 of the host protein. DLL is not involved. It is possible that SesJ binds plasminogen by a similar mechanism. Plasminogen captured on the bacterial cell surface could be activated to the potent serine protease plasmin by host plasminogen activators. This could contribute to pathogenesis, for example, by degrading opsonins. A more exhaustive search for potential ligands including those that bind *S. aureus* MSCRAMMs such as cytokeratins, loricrin, and elastin and investigating a role in biofilm formation is warranted.

#### SesI

The CWA protein SesI is small and has no distinguishing features. The presence of the *sesI* gene is associated with disease causing isolates and less so with commensals isolated from the skin or nares (Qi et al., 2017). The majority of *sesI* carrying strains were from ST2. A *sesI* defective mutant of RP62a was slightly less adhesive to an abiotic surface and planktonic cells formed fewer clumps compared to wild type. However, the density of biofilm formed by the mutant under static growth conditions *in vitro* was no different to that of the wild type. The mutant was not tested in a biofilm infection model. The association of SesI with nosocomial isolates could be coincidental.

## Discussion

The genome sequences of more than 1,000 clinical and commensal skin isolates of *S. epidermidis* have recently become available (Meric et al., 2018; Espadinha et al., 2019; Zhou et al., 2020). As more strains were sequenced the core genome shrank and the accessory genome expanded revealing that *S. epidermidis* is open to gene exchange. A detailed genome wide association study (GWAS) failed to determine if any surface protein is associated with disease isolates although polymorphisms within some surface protein genes may be involved (Meric et al., 2018). A study focussing on CWA proteins is warranted.

The genes encoding the SesJ protein and its associated glycosyl transferases are located within SCC mobile genetic elements (Arora et al., 2020). The *sesJ* gene is present in about 18% of disease isolates from several different STs including ST2 that were isolated in two American hospitals. However, the SCC*mec* type IV element carrying *sesJ* is not present in the globally disseminated ST2 strains which instead harbor a SCC*mec* type III element (Lee et al., 2018). The *sesJ* gene was also present in a small collection of commensal isolates. A detailed analysis of clinical and commensal isolates from diverse sources will shed light on any possible association of SesJ with disease although the comprehensive GWAS analysis mentioned above did not report any such link (Meric et al., 2018).

The identification of amyloid fibers as integral components of biofilm matrices is a significant development in understanding the complexities of biofilm formation. Initially the formation of amyloids by PSMs in *S. aureus* was suggested to be of importance (Schwartz et al., 2012) but this proved to be controversial when studies with *S. epidermidis* did not concur (Zheng et al., 2018).

The ubiquitous small basic protein (Sbp) has an important role in biofilm formation (Decker et al., 2015). The Sbp protein is secreted as a soluble monomer and can readily form amyloid fibrils *in vitro* (Wang et al., 2018). Sbp amyloids are integral components of the biofilm matrix formed by cells expressing both PIA/PNAG and Aap (Decker et al., 2015). The Aap protein and its homologue SasG in *S. aureus* trigger biofilm accumulation by homophilic Zn^2+^-dependent twisted rope interactions (Conrady et al., 2013; Formosa-Dague et al., 2016). As the biofilm matures Aap proteins form amyloid fibrils (Yarawsky et al., 2020). The structures of Sbp and Aap amyloids await elucidation.

Prosthetic bone and joint infections often require surgical intervention because treatment with antibiotics alone is inadequate due to many of the bacteria being in a semi-dormant or persister state. Novel therapies with phage lysins, biofilm matrix degrading enzymes, and quorum sensing blockers have been proposed (reviewed by Otto, 2018). Inhibition of the Agr quorum sensing system might reduce dissemination of cells from the infected device due to reduced expression of PSMs but this may not prevent with the establishment of biofilm (Le et al., 2019).

Monocytes that are associated with biofilm infections are polarized toward an anti-inflammatory state (Le et al., 2018; Yamada et al., 2020). Directed uptake by monocytes of nanoparticles coated with the mitochondrial oxidative phosphate inhibitor oligomycin reprogrammed metabolism toward pro-inflammatory glycolysis (Yamada et al., 2020). These macrophages reduced biofilm density *in vitro* and *in vivo* in a mouse model of biomaterial-associated bone infection. Combined with antibiotics, this led to sterilization of the infected implant. This study was performed with *S. aureus* but it is worthwhile speculating that this treatment will also reduce *S. epidermidis* prosthetic joint infection.

Significant advances have been made in understanding the mechanistic basis of cell-cell accumulation during biofilm formation. The importance of the Sbp in both PIA and Aap promoted biofilm and its formation of amyloids, as well as amyloid formation during the late accumulation phase by Aap indicates an additional layer of complexity. Prevention of biofilm formation on indwelling devices and disruption of established biofilm is a goal for future studies. GWAS studies identified several factors that are associated with disease causing strains but key factors that could be targeted for intervention were not apparent.

## Author Contributions

The author confirms being the sole contributor of this work and has approved it for publication.

### Conflict of Interest

The author declares that the research was conducted in the absence of any commercial or financial relationships that could be construed as a potential conflict of interest.
